# Setting up minimal invasive surgery services in gynecology in a resource-limited setting: an experience from Bhutan

**DOI:** 10.1186/s13104-022-05953-0

**Published:** 2022-02-16

**Authors:** Sangay Tshering, Thinley Dorji, Namkha Dorji, Renuka Monger, Kesang Choden, Kezang Lhamo

**Affiliations:** 1 Department of Obstetrics & Gynecology, Jigme Dorji Wangchuck National Referral Hospital, Thimphu, Bhutan; 2 Department of Internal Medicine, Jigme Dorji Wangchuck National Referral Hospital, Thimphu, Bhutan; 3 Kidu Mobile Medical Unit, His Majesty’s People’s Project, Thimphu, Bhutan

**Keywords:** Minimal invasive surgery, Gynecology, Bhutan

## Abstract

**Objective:**

To describe the clinical profile of minimal invasive procedures performed in gynecology at the national referral hospital in Bhutan. A review of such procedures performed in gynecology was needed to assess the baseline information and generate our own experience. We conducted a descriptive study with a review of hospital records of minimal invasive procedures performed from 1st January to 31st December 2020 at the Department of Gynecology. Data were extracted into a structured pro forma. Descriptive statistics were used to express the results.

**Results:**

The mean age of the patients was 33.9 ± 8.6 years of which the maximum was in the age group 25–34 years. 28 (17.5%) and 132 (82.5%) patients underwent emergency and elective procedures respectively. 142 (88.8%) and 18 (11.2%) patients underwent laparoscopic and hysteroscopic procedures respectively. Ovarian cystectomy was the most commonly performed procedure. The median operating time was 100 min (IQR 62.5–157.5). The overall complication rate was 2.5%. The median postoperative length of hospital stay was 24 h (IQR 3–24). Using our data and experience, we recommend a new health policy to recognize MIS in gynecology as a subspecialty and strengthen the existing service in gynecological MIS.

## Introduction

Surgical services are an essential component of health systems but facilities and trained human resources are often lacking in many resource-limited countries [1]. Gynecological conditions are a major contributor to morbidity and mortality worldwide, with the greatest burden of disease borne by women in resource-limited countries where access to specialists and surgical services is limited [2]. In the field of women’s health, much of the global progress has been achieved in the field of reducing maternal mortality while mortality due to gynecological conditions such as cancer of the cervix, ovaries, and uterus has remained unchecked. Overall, 4.5% of the global burden of disease is attributed to gynecological origin [2].

Benign gynecological diseases, abnormal uterine bleeding, reproductive tract infections, and urogynecological conditions constitute significant morbidity in low-resource settings and have a major impact on the quality of life of women [2]. A proportion of these diseases can be treated with surgeries and laparoscopic surgeries play a major role in reducing perioperative morbidity and mortality as well as reducing the overall cost of surgeries in resource-limited settings [2, 3]. However, resource-limited countries face significant challenges in providing laparoscopic surgeries—lack of opportunities for training of human resources, lack of stable financing sources, and lack supportive clinical care policies [4].

Laparoscopic surgeries, also known as minimal invasive surgery (MIS) are now the standard of care in many centers across the world [4]. It has shown benefits in terms of reduced postoperative pain, small scar, less post-operative hospital stay, and early return to work comparable to the Enhanced Recovery After Surgery (ERAS) protocol [5, 6]. With the improvement in MIS techniques, the newer generation of equipment, and the availability of adequate human resources, the rates of these procedures are on the rise [5, 7].

## Main text

### Study setting and design

In Bhutan, surgical services were institutionalized in the 1980s at the Jigme Dorji Wangchuck National Referral Hospital (JDWNRH) [8]. At our hospital, the scope of surgical services has gradually expanded while surgical services are now available in five other surgical centers across the country [9]. In 2019, the Department of Gynecology initiated MIS in many gynecological conditions which were otherwise performed with open surgeries. This study was planned to describe the clinical profile of MIS in gynecology at the Department of Gynecology, JDWNRH, Thimphu in its first year of operations in 2020. This was a descriptive study with a review of hospital records of gynecological surgeries performed during the one year, 1st January–31st December 2020. All patients who underwent MIS at the department were considered during the study period. The volume, types, and indications for procedures performed, duration of the procedure and postoperative hospital stay, and outcome of the procedures were analyzed in this study.

The Himalayan kingdom of Bhutan has a population of 0.7 million with a female population of 346,692 in 2017 [10]. Bhutan has a three-tiered health system with Basic Health Units at the primary level, district and general hospitals at the secondary level, and three referral hospitals at the tertiary level [11]. In 2020, Bhutan had 12 obstetrician-gynecologists practicing across the country. Patients with gynecological conditions can access specialist services at the three tertiary hospitals in Thimphu, Monggar, and Gelegphu and three other Emergency Obstetric Centers in Samtse, Phuentsholing, and Trashigang Hospitals. The JDWNRH is the largest hospital in the country with 381 beds [12]. The Department of Gynecology has 36 beds staffed with six obstetrician-gynecologists. Only two gynecologists completed short-term training in MIS. The hospital’s operation room (OR) schedule allocates one day per week for elective minimal invasive gynecological surgeries.

### Data entry and analysis

Data variables were listed according to the study objectives. All data were extracted from the electronic database maintained at the OR using the search word “lap/hysteroscopic” using the filter option. The admission register maintained at the gynecology ward was physically searched for patients’names and type of procedure performed. The patient files were then retrieved from the hospital’s medical record section. Data information was extracted using a structured proforma by the investigators. Duplication of patients from readmission to the hospital within one month of surgery was verified through careful evaluation of the patient’s name, citizenship identity card, or hospital registration number and deleted from the final dataset. This provided the data on readmission following any complication from the primary surgery. Any repeat additional procedure for a separate indication was counted as a separate procedure. Data was double entered between January and March 2021, validated and analyzed using EpiData (version 3.1 for entry and version 2.2.2.183 for analysis, EpiData Association, Odense, Denmark). Additional analysis was performed using STATA (version 13.0, StataCorp LP USA). For categorical variables, data were expressed as frequencies and percentages. For continuous variables, data were expressed as mean (Standard Deviation) and median (Inter Quartile Range).

### Results

A total of 1170 gynecological patients were operated on during the year 2020. 160 patients availed the services in MIS in gynecology during the study period. The surgical volume for MIS in gynecology was 13.7%. The mean age of the patients was 33.89 ± 8.58 years of which the maximum was in the age group 25–34 years. 28 (17.5%) and 132 (82.5%) patients underwent emergency and elective procedures respectively. 142 (88.8%) and 18 (11.2%) patients underwent laparoscopic and hysteroscopic procedures respectively. 109 patients underwent minimal invasive procedures with therapeutic intent and the remainder 51 with diagnostic intent.

The types of minimal invasive procedures performed is presented in Table 1.

**Table 1:** Distribution of types of minimal invasive gynecological procedures performed

Sl no	Types of minimal invasive procedures performed	Frequency (*n* = *160*)	Percentage (%)
1	Salpingectomy	41	25.6
2	Salpingo-oophorectomy	6	3.8
3	Adhesiolysis	13	8.0
4	Ovarian cystectomy	41	25.6
5	Uterine septal resection	4	2.5
6	Total laparoscopic hysterectomy(TLH)	4	2.5
7	Diagnostic laparoscopy	37	23.1
8	Diagnostic hysteroscopy	4	2.5
9	Others	10	6.4

**Fig. 1:**
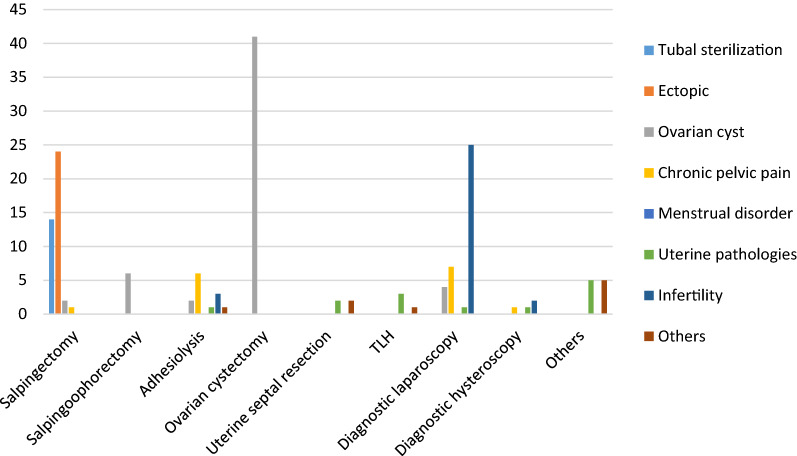
Types of minimal access gynecological surgeries against the indications

Based on the single indication, ovarian cystectomy was the most commonly performed surgical procedure followed by salpingectomy (Fig. 1).

The median operating time was 100 min (62.5–157.5) and the maximum number of procedures (44.4%) took between 60 and 119 min (Table 2). There were three conversions to laparotomy giving a 1.9% conversion rate. The overall complication rate was 2.5%. The median duration of postoperative hospital stay was calculated at 24 h ( IQR 3–24).

**Table 2:** Duration of operating time against the number of procedures performed

Sl No	Duration of surgery (in minutes)	Frequency (n = 160)	Percentage (%)
1	< 30	13	8.1
2	30–59	26	16.3
3	60–119	71	44.4
4	≥ 120	50	31.2

### Discussion

Currently, the services in MIS are limited to simple diagnostic and therapeutic procedures. Studies from resource-limited countries reported similar findings in terms of indications and type of procedure performed as they initiated gynecological minimal invasive procedures [13–15]. In our study, the commonly performed procedures were ovarian cystectomy, salpingectomy, and diagnostic laparoscopy in the age group 25–44 years. This is in agreement with other studies as most of the benign ovarian pathologies, ectopic and reproductive issues occur in this age group [14, 16, 17]. The moderate to complex procedures like ovarian cystectomy and TLH contributed to a longer operating time. In our study, 71% of the ovarian cystectomy and 100% of TLH took more than 120 min to complete the procedure. This indirectly points to low surgical volume leading to a slow learning curve and experience of the operating surgeon [18–20]. While we agree on these personal characteristics for longer operating time, ergonomics plays an equally important contributory role. There are well-established studies to relate occupational-related long-term health hazards especially chronic pain syndromes in laparoscopic procedures resulting from poor ergonomics [21–23]. As mentioned by Sangtaeck et al., we also experienced technical difficulty in performing intracorporeal suturing in TLH leading to prolonged operating time over four hours [24]. Two ovarian cystectomies and one TLH were converted to laparotomy due to uncontrolled bleeding and camera-head troubleshooting respectively. Classified as grade II complications based on Clavien Dindo classification, four ovarian cystectomies needed an unexpected blood transfusion during the intraoperative period. The overall complication rate of 2.5% was compatible with the findings by Fuentes et al. based on the degree of operative difficulty [7]. There were no major intestinal or vascular complications in our study.

As we strive to widen the scope of MIS in gynecology, this study will serve as the baseline data for our hospital. As evident in resource-limited settings, there were barriers to the practice of minimal invasive gynecological surgery. Limited clinical exposure and training, lack of dedicated scrub nurses and OR, inadequate instruments and maintenance, backlog, and time pressure are some of the challenges in our setup [3, 4]. From a resource-limited setting point of view, we hereby share our experience encountered while setting up and performing MIS in gynecology.

The role of MIS in reducing overall health care costs is well documented in several studies. Barnett et al. found out that MIS was the least expensive compared to robotic and laparotomy while treating endometrial cancer [25]. This is of paramount importance as Bhutan provides free healthcare to its citizens against the ever-rising cost of health care [10, 11]. The median postoperative length of hospital stay of 24 h in our study is lower compared to the usual 72 h for laparotomy which converts to reduced health care expenditure and addressing bed shortage in our setting.

Apart from JDWNRH, the two regional referral hospitals in the country also provide limited MIS in gynecology. Tubal sterilization and a few diagnostic procedures are currently provided at the regional hospitals. As a result, the number of patients wishing to undergo these procedures at the national referral hospital is ever increasing. This has overburdened the two gynecologists who completed formal short-term training in MIS. Lack of trained and dedicated scrub nurses also hamper the availability of 24-h services in MIS in gynecology. Frequent breakdown of equipment and troubleshooting has led to the cancellation of procedures as the scrub nurses are not trained to deal with such problems. Routine cases are performed only once a week as we have to share the equipment and scrub nurses with the surgical department. Moreover, odd-hour emergency minimal invasive procedures are not performed due to a lack of trained gynecologists and scrub nurses on duty. All these factors have created a huge backlog of cases and time pressure extending beyond three months on average.

Lack of basic equipment like tissue retrieval bags, morcellator, resectoscope, a uterine manipulator with colpotomizer, and limited energy sources prolong the operating time and make procedures technically challenging. In the absence of a hysteroscopic infusion pump, operative hysteroscopy is limited to uterine septal resection and small endometrial polypectomy using hysteroscopic scissors. The hospital does not have a dedicated OR to perform minimal invasive procedures resulting in frequent mobilization of equipment from one room to another. The single monitor screen, manually adjusting OR table with fixed candy cane leg stirrups also contribute to poor ergonomics and longer operating time.

The department has initiated an inter-institutional collaboration to educate and train the gynecologists and residents in MIS in gynecology. A basic low-cost simulation laboratory was set up to train the gynecologists and residents. As the cost factor is a bottleneck in initiating and sustaining the minimal invasive procedures, we reused all the disposable equipment after high-level disinfection. We also customized the tissue retrieval bags using a latex condom, pediatric urine bag, and surgical gloves. Larger tissues are cut into smaller pieces and removed through a 10 mm port using a gall bladder extractor and gall bladder stone forceps. In hysteroscopic procedures, less than two liters of normal saline is manually pumped to distend the uterus. The candy cane leg stirrups are padded with cotton and positioned at around 120 degrees against the edge of the operating table. Extracorporeal knot tying is performed in cases of hysterectomy as it is technically easier compared to intracorporeal suturing and knot tying.

The numerous benefits of MIS in gynecology to the patient, hospital, and health system at large cannot be overemphasized. At the institutional level, we recommend training scrub nurses to be fully dedicated to these procedures. More gynecologists need to be trained in this field and services cascaded down to the regional hospitals. Without a policy to recognize MIS in gynecology as a subspecialty, it would be difficult to maintain and strengthen this service at the national referral and regional hospitals. Upcoming infertility and gyne-oncology center as a part of the multidisciplinary hospital would benefit tremendously if a separate health policy is framed to address health financing and budgeting for MIS in gynecology in the future.

### Conclusion

The formal introduction of MIS in gynecology at our hospital has benefitted the patients and health system at large. As in any resource-limited setting, we faced challenges in initiating and performing these procedures in gynecology. We also adopted and adapted technical aspects while performing the procedures. Using our data and experience, we recommend a new health policy to recognize MIS in gynecology as a subspecialty and strengthen the existing service in gynecological MIS.

### Limitations

This was the first hospital-based study done on MIS in gynecology in Bhutan. As such, the study provides the baseline information on surgical volume, indications, types of procedures performed, and the outcome. Since this was a single-center review of records, the results generated from our study cannot be generalized.

## Data Availability

The data set analyzed in this study is available with the corresponding author upon request.

## Abbreviations

MAS: Minimal invasive surgery
JDWNRH: Jigme Dorji Wangchuck National Referral Hospital
OR: Operation room
TLH: Total laparoscopic hysterectomy
